# Pharmacological targeting of ferroptosis in hypoxia-induced pulmonary edema: therapeutic potential of ginsenoside Rg3 through activation of the PI3K/AKT pathway

**DOI:** 10.3389/fphar.2025.1644436

**Published:** 2025-07-22

**Authors:** Yacong He, Yilang Wang, Huxinyue Duan, Demei Huang, Nan Jia, Zherui Shen, Zhenxing Wang, Mingjie Wang, Tianzhu Zhao

**Affiliations:** ^1^ State Key Laboratory of Southwestern Chinese Medicine Resources, School of Pharmacy, Chengdu University of Traditional Chinese Medicine, Chengdu, China; ^2^ Teaching and Research Office of Traditional Chinese Medicine Internal Medicine, Hospital of Chengdu University of Traditional Chinese Medicine, Chengdu, China; ^3^ School of clinical Medicine, Chengdu University of Traditional Chinese Medicine, Chengdu, China; ^4^ School of Basic Medical Sciences, Chongqing University of Chinese Medicine, Chongqing, China; ^5^ Respiratory and Critical Care Medicine Center, Traditional Chinese Medicine Hospital of Meishan, Meishan, China

**Keywords:** high-altitude pulmonary edema (HAPE), ginsenoside Rg3 (G-Rg3), PI3K/Akt pathway, ferroptosis, hypoxic pulmonary hypertension

## Abstract

**Background:**

High-altitude pulmonary edema (HAPE), a severe manifestation of hypoxia-induced pulmonary hypertension, continues to present a major health concern in high-altitude environments due to the absence of efficient preventive measures. This investigation explores the protective influence of ginsenoside Rg3 (G-Rg3), an active substance derived from the botanical drug *Panax ginseng* C.A.Mey., on the prevention of HAPE progression.

**Methods:**

A mouse model mimicking exposure to 6000-m altitude (n = 63 C57BL/6 mice) was employed to evaluate the impact of G-Rg3 (15/30 mg/kg) using histopathological, biochemical, and multi-dimensional molecular assessments. Western blotting, network pharmacology and computational simulations were utilized to identify molecular targets of G-Rg3. The role of the PI3K/AKT signaling pathway was further validated through experiments using the PI3K/AKT inhibitor LY294002.

**Results:**

Pre-treatment with G-Rg3 effectively alleviated HAPE, maintained the stability of lung ultrastructure, and inhibited inflammatory mediators and oxidative stress indicators. Mechanistically, G-Rg3 prevented ferroptosis by stimulating the PI3K/AKT signaling pathway, as evidenced by the upregulation of protective proteins (GPX4, Nrf2, HO-1, SLC7A11, FTH1, FLC) and the downregulation of iron metabolism regulatory factors (TFRC, COX2). Network pharmacology and molecular docking analysis confirmed that PI3K/AKT is the core target of G-Rg3, and the protective effect disappeared when this pathway was inhibited. G-Rg3 uniquely regulated oxidative stress and inflammation by inhibiting ferroptosis, demonstrating adaptability to high-altitude environments.

**Conclusion:**

This research examined the pharmacological impacts and molecular pathways of ginseng active monomers on HAPE, suggesting the potential of G-Rg3 as a promising treatment option for this condition.

## 1 Introduction

High-altitude pulmonary edema (HAPE) represents a critical form of hypoxic pulmonary hypertension, typically emerging in settings with diminished atmospheric pressure and oxygen levels (Bärtsch and Swenson, 2013; Persson and Bondke Persson, 2017). Extended exposure to high-altitude hypoxia is the primary driver of HAPE, which, without intervention, can evolve into chronic hypoxic pulmonary hypertension. Clinically, HAPE is marked by non-cardiogenic pulmonary edema and worsening hypoxemia, with an untreated fatality rate estimated at nearly 50% (Bouzat et al., 2013; Li et al., 2024; Menon, 1965). Individual susceptibility to hypoxic stress and the development of HAPE may be influenced by genetic variations, particularly in mitochondrial DNA and components of the renin-angiotensin-aldosterone system (RAAS) (Bhagi et al., 2015; Luo et al., 2012; Sharma et al., 2019; Srivastava et al., 2012). Furthermore, hypoxic conditions can induce oxidative stress via mitochondrial dysfunction and disruptions in metabolic pathways, thereby amplifying abnormalities in the pulmonary vasculature (Ali et al., 2012; Sharma et al., 2021). Importantly, in high-altitude environments, cold temperatures may interact synergistically with hypoxia to exacerbate cardiopulmonary dysfunction, thus elevating the risk of HAPE (Eichstaedt et al., 2023). These underlying pathophysiological mechanisms ultimately result in abnormal pulmonary vascular reactions and sustained pulmonary hypertension, leading to the formation of edema.

In recent years, there has been a growing emphasis on the use of natural medicines for treating HAPE, particularly those derived from botanical drugs with lung-protective active metabolites. Recent clinical studies have highlighted the significant potential of traditional botanical drugs, such as ginseng, in preventing and managing HAPE through multi-target mechanisms (Huang et al., 2024; Shen et al., 2023). *Panax ginseng* C.A.Mey. (known as Renshen in Chinese), a classic botanical drug historically used for respiratory enhancement, exemplifies this potential through its anti-inflammatory, antioxidant, and cellular protective properties (Ratan et al., 2021). One notable metabolite, ginsenoside Rg3 (G-Rg3), demonstrates potential efficacy in mitigating hypoxic lung injury (Bae et al., 2014; Chen et al., 2010; Wang et al., 2016). Its mechanism of action involves promoting cell survival by activating the PI3K/AKT signaling pathway (Yang et al., 2018). Our previous study demonstrated that intraperitoneal administration of G-Rg3 (15/30 mg/kg) significantly mitigated acute mountain sickness (AMS) in C57BL/6 mice through ferroptosis regulation (Liu et al., 2025a). These advancements not only underscore the scientific significance of traditional medicinal resources but also offer a theoretical foundation for developing plant-based therapies against high-altitude hypoxia.

Oxidative stress plays a central role in the pathogenesis of HAPE. Under normoxic conditions, endogenous antioxidant systems, including catalase, glutathione (GSH), and superoxide dismutase (SOD), maintain redox balance by neutralizing reactive oxygen species (ROS) (Diaz-Vivancos et al., 2015; Sies, 2017). Hypoxia disrupts this balance through two main mechanisms: impaired electron transport chain function due to oxygen deficiency increases ROS production (Gaur et al., 2021), while simultaneous depletion of antioxidant reserves exacerbates oxidative damage to lipids, proteins, and DNA (Wang et al., 2022b). This dual disruption creates a self-perpetuating cycle of oxidative injury, eventually overwhelming cellular repair mechanisms and triggering redox imbalance (Yu et al., 2021).

The resulting oxidative stress induces ferroptosis, an iron-dependent form of programmed cell death marked by three key features: (1) membrane rupture via lipid peroxidation cascades, (2) intracellular iron overload, and (3) failure of the glutathione peroxidase 4 (GPX4) system (Dixon et al., 2012; Yang and Stockwell, 2016). Mechanistically, hypoxia-induced ROS overproduction initiates lipid peroxidation through Fenton reactions, while iron accumulation amplifies oxidative damage by catalyzing hydroxyl radical formation (Djulbegovic and Uversky, 2019). These processes compromise membrane integrity and inactivate iron-regulatory proteins, establishing a pathological feedback loop. Recent evidence links ferroptosis to the activation of Phosphoinositide 3-kinase/Protein Kinase B (PI3K/AKT) and Mitogen-Activated Protein Kinase (MAPK) signaling pathways, which are involved in both cellular survival decisions and hypoxic adaptation (Gao et al., 2025; Guo et al., 2022). Notably, preclinical models demonstrate ferroptosis involvement in hypoxia-induced organ damage, including neurological impairment following acute altitude exposure (Han et al., 2025), suggesting its potential as a therapeutic target in HAPE pathophysiology.

In view of the aforementioned research context, our study puts forward the hypothesis that G-Rg3 may prevent HAPE by suppressing ferroptosis. To verify this hypothesis, we developed a HAPE model induced by hypobaric hypoxia in C57BL/6 mice. The therapeutic efficacy of G-Rg3 on HAPE was assessed using multiple approaches, including quantitative analysis of pulmonary edema, histopathological scoring, profiling of inflammatory cytokines, and evaluation of oxidative stress biomarkers. To investigate the mechanistic regulation of ferroptosis by G-Rg3, we employed an integrated strategy combining computational techniques (network pharmacology, molecular docking, and molecular dynamics simulations) with experimental validation methods: immunofluorescence detection of ferroptosis markers, transmission electron microscopy for mitochondrial ultrastructure analysis, and Western blot examination of PI3K/AKT signaling pathway components.

This holistic approach not only facilitates the identification of therapeutic targets involved in ferroptosis-driven progression of HAPE but also establishes a theoretical foundation for G-Rg3 as a promising candidate for treating this condition.

## 2 Materials and methods

### 2.1 Materials

Ginsenoside Rg3 (G-Rg3, ≥99.15% purity, Cat.14197-60-5) was procured from Must Biotechnology (Chengdu, China). The PI3K/AKT pathway inhibitor LY294002 (Cat.S1105) and all analytical-grade chemicals were obtained from Selleck Chemicals (United States) and commercial suppliers, respectively. Oxidative stress parameters were measured using Nanjing Jiancheng kits: Malondialdehyde (MDA, A003-1), GSH (A006-2-1), SOD (A001-3), and tissue iron quantification (A039-2-1). Proinflammatory cytokines Interleukin-1 beta (IL-1β, ZC-37974W), Interleukin 6 (IL-6, ZC-37988W), and Tumor necrosis factor-alpha (TNF-α, ZC-39024W) were analyzed with Zhucai Biotechnology ELISA kits (Shanghai).

Antibody Specifications Immunoblotting employed the following primary antibodies: Abcam (UK): Vascular Endothelial Growth Factor (VEGF, ab32152), Hypoxia-inducible factor 1 alpha (HIF-1α, ab179483), Nuclear Factor Erythroid 2-Related Factor 2 (Nrf2, ab92946), Heme Oxygenase-1 (HO-1, ab68477), GPX4 (ab125066), Ferritin light chain (FLC, ab75973), Transferrin receptor gene (TFRC, ab269513), PI3K/AKT pathway components (PI3K ab191606, AKT ab185633, p-PI3K ab182651, p-AKT ab192623), β-actin (ab227387). Cell Signaling Technology (United States): Ferritin Heavy Chain 1 (FTH1, #4393S), Cyclooxygenase-2 (COX2, #12282S). ABclonal (Wuhan): Solute Carrier Family 7 Member 11 (SLC7A11, A2413) Secondary detection used HRP-conjugated goat anti-rabbit IgG (BF03008, Biodragon Biotech).

All supplementary chemicals meeting analytical-grade specifications.

### 2.2 Animals

Sixty-three male C57BL/6 mice (6–8 weeks, 18–22 g, specific pathogen-free) were sourced from Chengdu Dasuo Biological Technology (Certification SCXK 2022–0345). Following 7-day acclimation under controlled conditions (22°C ± 1°C, 55% ± 5% humidity; 12-hr light/dark cycle), animals received standardized feeding with autoclaved water and chow (Zhang et al., 2025a). The study received ethical approval (2022-18) from Chengdu University of Traditional Chinese Medicine’s Animal Welfare Experimental Center, with all procedures complying with institutional ethics guidelines and national welfare legislation.

### 2.3 Experimental design

Animals were randomly divided into five experimental subgroups (n = 7 per group): (1) Sham control, (2) G-Rg3 monotherapy (30 mg/kg), (3) HAPE model, (4) HAPE + G-Rg3-L (15 mg/kg), and (5) HAPE + G-Rg3-H (30 mg/kg), with G-Rg3 dosages validated by prior pharmacological studies (Cheng and Li, 2016; Heinrich et al., 2020; Liu et al., 2025a). While Sham and HAPE groups received intraperitoneal (*i.p.*) phosphate-buffered saline (PBS), other cohorts were administered G-Rg3 via *i. p*. injection for 72 consecutive days. To investigate PI3K/AKT-ferroptosis regulatory mechanisms, an additional cohort of 28 mice underwent randomization into four groups: (1) HAPE baseline, (2) HAPE + G-Rg3-H (30 mg/kg), (3) HAPE + LY294002 (5 mg/kg PI3K/AKT inhibitor), and (4) HAPE + G-Rg3-H/LY294002 combinatorial therapy. LY294002 formulations utilized a solvent system containing 50% distilled water, 40% PEG300, 5% Tween 80, and 5% DMSO. All interventions employed standardized i. p. administration protocols (5 mL/kg injection volume, daily dosing over 3 days).

### 2.4 HAPE modelling

To replicate the HAPE condition in mice, we employed a hypobaric hypoxic chamber model ProOx-830 from Tawang Intelligent Technology (Shanghai, China), following the protocols outlined in our previous research and relevant publications (Ma et al., 2020; Tan et al., 2020; Wang et al., 2022b). After a 3-day administration period, the mice were transferred to a hypobaric hypoxia chamber set at an altitude of 6000 m, with an oxygen partial pressure of 9.6 kPa, humidity of 60%, and temperature of 20°C. The animals were rapidly elevated to this altitude within 5 min at a rate of 20 m per second and maintained there for 48 h.

**FIGURE 1:**
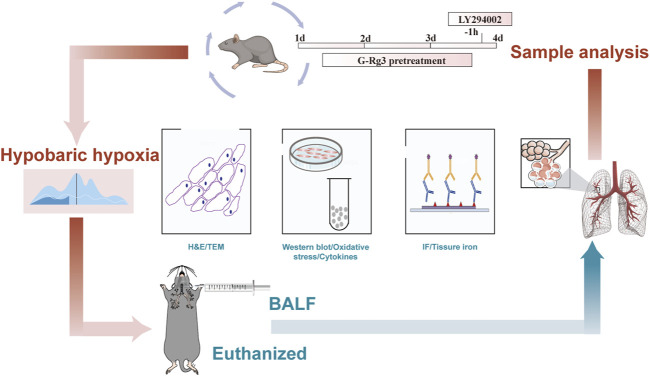
The flowchart of HAPE animal experiments.

Following the 48-h modeling period, the altitude was gradually adjusted to normal levels, and then the mice were taken out of the chamber and euthanized via an i. p. injection of sodium pentobarbital. Following euthanasia, blood was collected from the abdominal aorta for serum extraction, bronchoalveolar lavage fluid (BALF) was obtained, and lung tissues were sectioned for additional analyses. The experimental protocol for the HAPE animal model is depicted in Figure 1.

### 2.5 Histology of lung tissue

Post-euthanasia pulmonary specimens underwent standardized histoprocessing: The right middle lung lobe was immediately harvested, cleared of extraneous tissue, and immersion-fixed in ice-cold 4% paraformaldehyde (12 h, 4°C). Sequential ethanol dehydration preceded paraffin embedding, with resultant blocks sectioned at 4 μm thickness. Following hematoxylin-eosin (H&E) staining, slides were imaged using an OLYMPUS BX41 microscope. Two professional pathologists blindly scored the degree of lung tissue damage using the McGuigan pathology scoring method (Faller et al., 2012; McGuigan et al., 2003).

### 2.6 Lung wet/dry (W/D) weight ratio

The right upper lung lobe was surgically harvested post-euthanasia for hydrostatic evaluation. Fresh tissue mass (W) was immediately recorded before dehydration at 60°C until mass stabilization (D), enabling calculation of the W/D ratio - a validated indicator of alveolar fluid accumulation severity in hypobaric pulmonary edema models.

### 2.7 Cytokine content in BALF

Following right lung ligation, the left lung underwent dual bronchoalveolar lavage procedures utilizing 0.2 mL ice-cold PBS administered via tracheal cannulation. Pooled lavage fluid was centrifuged (12,000×g, 10 min, 4°C) to separate cellular components, with the resultant supernatant aliquoted for cryopreservation at −80°C pending cytokine analysis. Quantitative assessment of IL-1β, IL-6, and TNF-α concentrations in BALF was conducted through enzyme-linked immunosorbent assay (ELISA) following manufacturer-specified protocols (Thermo Fisher Scientific).

### 2.8 Oxidative stress and iron content in lung tissues

The right posterior lobe of the lung was surgically removed and perfused with sterile saline prior to mechanical homogenization in PBS. Cellular debris was eliminated by centrifugation at 12,000×g for 15 min at 4°C, allowing us to collect clear supernatants for further biochemical evaluation. Key markers of oxidative stress, including MDA levels, SOD activity, and GSH concentrations, as well as parameters related to iron metabolism, were quantified using commercially available assay kits. Absorbance values were measured using a Thermo Scientific Varioskan LUX microplate reader. To ensure accurate quantitative comparisons across samples, total protein content was normalized using the bicinchoninic acid (BCA) protein assay method.

### 2.9 Western blotting

The right inferior pulmonary lobe was immediately excised from euthanized mice and flash-frozen in liquid nitrogen prior to cryostorage at −80°C for subsequent molecular characterization. Tissue homogenates prepared in RIPA buffer containing protease/phosphatase inhibitors (1 mM PMSF) underwent centrifugation (12,000×g, 10 min, 4°C), with supernatants subjected to BCA protein quantification (Pierce Biotechnology) per manufacturer specifications. Aliquots (30 μg) underwent electrophoretic separation on 8%–15% gradient SDS-PAGE gels (Bio-Rad Laboratories) followed by semi-dry transfer to PVDF membranes. Post-blocking with 5% BSA/TBST (1 h, 25°C), membranes were probed with primary antibodies at 4°C (16 h) targeting: VEGF (1:2000), HIF-1α (1:1000), Nrf2 (1:1000), GPX4 (1:5000), HO-1 (1:10,000), FLC (1:1000), TFRC (1:5000), PI3K (1:1000), AKT (1:2000), p-PI3K (1:800), p-AKT (1:1000), FTH1 (1:1000), COX2 (1:1000), SLC7A11 (1:2000) and β-actin (1:10,000)as normalization control. Post-TBST washes, HRP-conjugated secondary antibodies (1:5000, 2 h, 25°C) enabled chemiluminescent detection via ECL substrate, with band visualization (GelView 6000Plus) and densitometric quantification (Image-Pro Plus 6.0) relative to β-actin expression.

### 2.10 Transmission electron microscopy (TEM)

The excised apex of the right pulmonary lobe underwent dual fixation protocol involving primary stabilization in 3% glutaraldehyde (24 h, 4°C) followed by secondary osmication with 1% osmium tetroxide (2 h, 25°C). Processed through acetone-gradient dehydration and epoxy resin (Epon 812) polymerization, ultrastructural specimens were microtomed at 70 nm thickness. Transmission electron microscopy was conducted using a JEOL JEM-1400-FLASH system operating at 120 kV, with contrast enhancement achieved through sequential uranyl acetate and lead citrate staining regimens.

### 2.11 Immunofluorescence staining

Paraffin-embedded lung tissue sections (4 μm) underwent sequential processing through xylene deparaffinization and graded alcohol rehydration. For antigen retrieval, heat-mediated treatment was performed in 0.01 M citrate buffer (pH 6.0) for 10 min. Subsequent pre-treatment included 15-min endogenous peroxidase blockade with 0.3% H_2_O_2_ and 20-min non-specific binding inhibition using 10% normal goat serum. Primary antibody incubation proceeded overnight at 4°C with anti-GPX4 (1:100) and anti-p-PI3K (1:100), followed by 30-min room temperature exposure to species-matched secondary antibodies. Nuclear counterstaining was achieved through 10-min DAPI application (1 μg/mL) at 25°C, with residual dye removal via triple PBS washing (5 min each). Fluorescent signal acquisition utilized a Nikon C2 confocal system (Tokyo, Japan), with subsequent quantitative analysis performed using ImageJ (NIH, United States) for fluorescence intensity measurements.

### 2.12 Network pharmacology

#### 2.12.1 Target genes acquisition

The identification of G-Rg3 target genes incorporated a multi-platform strategy utilizing both computational prediction tools and pharmacological databases. Initial screening was performed through the Encyclopedia of Traditional Chinese Medicine (ETCM, http://www.tcmip.cn/ETCM), TargetNet (http://targetnet.scbdd.com), Swiss Target Prediction (http://www.swisstargetprediction.ch), and PharmMapper (http://www.lilab-ecust.cn/pharmmapper). To compensate for potential database limitations, literature-curated targets from prior ligand-receptor interaction studies were integrated (Wang et al., 2022a). Concurrently, HAPE-associated genes were systematically collated from four disease-specific repositories: GeneCards (https://www.genecards.org), OMIM (https://omim.org), CTD (http://ctdbase.org), and DisGeNET (http://www.disgenet.org). All identified genes underwent nomenclature standardization via a bioinformatics pipeline combining UniProt (https://www.uniprot.org) for sequence annotation and STRING (https://string-db.org) for orthology mapping, ensuring compliance with HUGO Gene Nomenclature Committee guidelines (Yin et al., 2024).

#### 2.12.2 Development of the protein-protein interaction (PPI) network

Following deduplication, gene set intersections between pharmacological targets and pathological associations were graphically represented through Venn diagram construction. Hub gene identification employed PPI network modeling via the STRING platform (organism: *Homo sapiens*; confidence threshold ≥0.7). The resultant interactome data underwent advanced topological analysis using Cytoscape v3.6.2, facilitating three-dimensional visualization of the metabolite-target-disease triad and systematic interrogation of network architecture through integrated bioinformatics tools.

#### 2.12.3 Gene pathway analysis

Following the identification of key genes in the network through topological analysis, a multi-level functional assessment was carried out using an integrated bioinformatics approach (with the clusterProfiler package). This framework executed Gene Ontology (GO) enrichment analysis for cellular process annotation and Kyoto Encyclopedia of Genes and Genomes (KEGG) pathway mapping to contextualize biological systems simultaneously. As a result, it enabled a comprehensive functional evaluation connecting molecular functions, biological processes, and pathways.

### 2.13 Molecular docking

#### 2.13.1 Molecular acquisition

The PI3Kγ structural coordinates for molecular docking were acquired from the RCSB Protein Data Bank (accession code 3ML9) (Burley et al., 2017), while the bioactive conformation of Ginsenoside Rg3 was obtained from PubChem (CID: 12855989) and energetically optimized through MMFF94 molecular mechanics refinement (Sunghwan et al., 2016).

Molecular docking utilized AutoDock Vina version 1.2, with receptor proteins preprocessed in PyMOL 2.5 to remove water, salt ions, and other small molecules beforehand (Eberhardt et al., 2021). The central coordinates for the docking box were determined by the center_of_mass.py plugin, using the active site as a reference, with each side of the box being 22.5 Å long. Moreover, all preprocessed small molecules and receptor proteins were altered into the PDBQT format necessary for docking with AutoDock Vina 1.2, utilizing ADFR Suite 1.0 (Pradeep et al., 2015). The global docking search exhaustiveness was configured to 32, whereas the remaining parameters were kept at their default values. The highest-ranked docking conformation was selected as the bound structure for follow-up molecular dynamics simulations in this investigation.

#### 2.13.2 Molecule dynamics

Informed by the docking results, complexes derived from small molecules and proteins served as the initial configurations for extensive molecular dynamics simulations, which were performed using the AMBER 22 software (Salomon-Ferrer et al., 2013). Prior to initiating the simulations, the partial charges of the small molecules were computed using the Antechamber module and the Hartree-Fock (HF) SCF/6-31G* method via the Gaussian 09 software (Frisch et al., 2009; Wang et al., 2005). For the small molecules, the GAFF2 force field was utilized, while the ff14SB force field was applied for the proteins (Maier et al., 2015; Wang J. et al., 2024; Wang X. et al., 2004). For each system configuration, the LEaP module was employed to add hydrogen atoms. A truncated octahedral TIP3P water box, with a 10 Å buffer around the system, was then positioned. Additionally, Na^+^/Cl^−^ ions were introduced to neutralize any charge imbalances (Mark and Nilsson, 2001). Ultimately, the topology and parameter files needed for the simulations were produced.

Using the AMBER 22 software package, molecular dynamics simulations were executed (Salomon-Ferrer et al., 2013). An energy minimization process was applied to the system before initiating the simulation that consisted of 2500 iterations of steepest descent, succeeded by a further 2500 iterations employing the conjugate gradient technique. Upon finishing the energy minimization, the system underwent a controlled temperature increase from 0 K to 298.15 K over 200 picoseconds at a uniform rate, maintaining a constant volume throughout this process. To ensure a homogeneous dispersion of solvent molecules within the solvent box and maintain a temperature of 298.15 K, an NVT ensemble simulation was performed for a duration of 500 picoseconds. Subsequently, to stabilize the system, an equilibrium simulation under NPT conditions was carried out for an additional 500 picoseconds throughout the entire system. The composite system underwent an extended simulation lasting 100 nanoseconds within an NPT ensemble, with periodic boundary conditions applied consistently. During this period, non-bonded interactions were truncated at a cutoff distance of 10 Å. Long-range electrostatic interactions were evaluated using the Particle Mesh Ewald (PME) method (Sagui and Darden, 1999). The SHAKE algorithm was utilized to fix the lengths of hydrogen bonds (Kräutler et al., 2015), while Langevin dynamics with with a collision frequency of γ = 2 ps^-1^ was used for temperature control (Larini et al., 2007). The pressure was maintained at 1 atm, integration time steps were established at 2 femtoseconds, and trajectory data were captured every 10 picoseconds for further analysis.

#### 2.13.3 MM/GBSA binding free energy calculation

The MM/GBSA method was applied to evaluate the binding free energy between the protein and ligand across all systems (Chen et al., 2020; Genheden and Ryde, 2015; Hou et al., 2011; Rastelli et al., 2010). Acknowledging that extended MD simulations can significantly affect the accuracy of MM/GBSA outcomes is crucial (Hou et al., 2011). Hence, this investigation used an MD trajectory covering 90–100 nanoseconds for the calculations, based on the formula described below:

$${\Delta G}_{bind}={\Delta G}_{\text{complex}}\;–\;\left({\Delta G}_{\text{receptor}}+{\Delta G}_{\text{ligand}}\right)$$

$$={\Delta E}_{\text{internal}}+{\Delta E}_{\text{VDW}}+{\Delta E}_{\text{elec}}{+\Delta G}_{\text{GB}}+{\Delta G}_{\text{SA}}$$

In the formula, internal energy is denoted by ΔE_internal_, van der Waals interaction by ΔE_VDW_, and electrostatic interaction by ΔE_elec_. The internal energies, which include bond energy (E_bond_), angle energy (E_angle_), and torsion energy (E_torsion_), are collectively known as solvation free energy. Here, ΔG_GB_ represents polar solvation free energy, while ΔG_SA_ denotes non-polar solvation free energy. In this study, the ΔG_GB_ model developed by Nguyen et al. (igb = 2) was utilized for calculations (Nguyen et al., 2013). To calculate the non-polar solvation free energy (ΔG_SA_), the surface tension (γ) was multiplied by the solvent-accessible surface area (ΔSASA), given by the formula ΔG_SA_ = 0.0072 × ΔSASA (Weiser et al., 1999). Entropy change calculations were not included in this study because they require significant computational resources and have low accuracy (Chen et al., 2020; Hou et al., 2011).

### 2.14 Statistical analyses

This research employed GraphPad Prism 8 (San Diego, California, United States) for data analysis and figure generation. For normally distributed datasets, one-way analysis of variance was conducted. The findings are expressed as mean ± SEM. A threshold of P < 0.05 was used to establish statistical significance.

## 3 Results

### 3.1 Effects of G-Rg3 pre-treatment on the prevention of HAPE

In instances of acute high-altitude pulmonary edema, patients typically exhibit clinical symptoms such as breathing difficulties, cyanosis of the mucous membranes, delayed reactions, and reduced activity levels (Gatterer et al., 2024). Consequently, we monitored the overall condition of mice to evaluate the pathological impacts of low-pressure hypoxia on the body, as well as the effectiveness of drug interventions. In their baseline state, all mice displayed normal physiological signs, characterized by glossy fur, steady respiration, and a heightened stress response. Following exposure to low-pressure hypoxia, distinct variations emerged among the groups: both the NC group and the G-Rg3 groups maintained normal activity levels, with regular breathing and feeding patterns; in contrast, the HAPE group demonstrated classic pathological features, including mucosal cyanosis, rapid breathing, and lethargic responses. The two dosage levels of G-Rg3 exhibited dose-related improvements, particularly the 30 mg/kg group, which showed markedly enhanced recovery in terms of activity, mucosal coloration, and respiratory rhythm.

**FIGURE 2:**
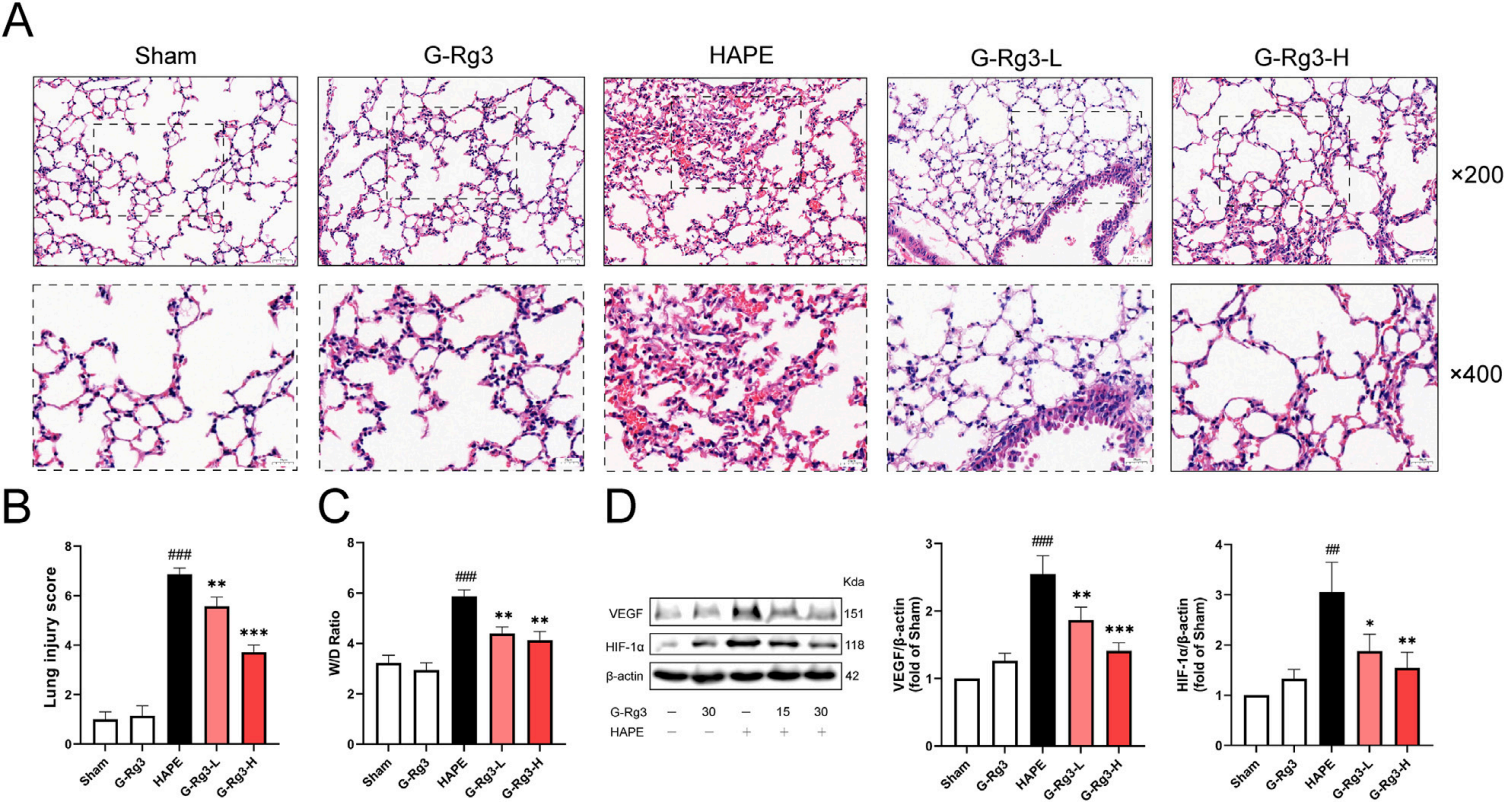
Effects of G-Rg3 pre-treatment on the prevention of HAPE. **(A)** H&E-stained lung sections (upper: ×200, 50 μm scale; lower: ×400, 20 μm scale). **(B)** Semi-quantified lung injury scores. **(C)** Hydrostatic imbalance via W/D weight ratio. **(D)** Hypoxic response markers VEGF and HIF-1α by immunoblotting. Data represent mean ± SEM (n = 6 biological replicates). One-way ANOVA with Tukey’s *post hoc* analysis: ^##/###^ *P* < 0.01/0.001 vs. Sham; ^*/**/***^ *P* < 0.05/0.01/0.001 vs. HAPE controls.

Histopathological analysis demonstrated that lung tissues in both the sham operation group and the G-Rg3 pre-treatment group remained structurally intact. Conversely, lung tissues in the HAPE group exhibited significant damage, including thickened alveolar walls, widened alveolar septa, alveolar cavity congestion, and prominent inflammatory cell infiltration (Figure 2A). These observations confirm the successful establishment of the rat model for high-altitude pulmonary edema. Following G-Rg3 pre-treatment, the extent of inflammatory cell infiltration, hemorrhage, and alveolar wall thickening was dose-dependently attenuated. Semi-quantitative assessment of lung tissue injury revealed that G-Rg3 pre-treatment markedly decreased the lung injury score associated with HAPE (Figure 2B) (McGuigan et al., 2003). Additionally, the wet/dry weight ratio of lung tissue was substantially elevated in the HAPE group compared to the sham operation group. However, this ratio was significantly lowered following G-Rg3 pre-treatment (Figure 2C).

As a pivotal transcription factor, HIF-1α modulates the expression of genes involved in hypoxic responses and is crucial for the physiological adaptation of organisms to hypoxic settings (Diaz-Vivancos et al., 2015). Elevated levels of HIF-1α have been conspicuously observed in individuals prone to HAPE under normoxic conditions. Moreover, empirical evidence indicates that HIF-1α contributes to the progression of HAPE through the regulation of its target gene, VEGF. Western blotting showed increased expression of VEGF and HIF-1α in the HAPE group, which was significantly attenuated following G-Rg3 pre-treatment (Figure 2D). The results showed that G-Rg3 pre-treatment could effectively reduce the inflammation and oxidative stress in the HAPE model.

### 3.2 Effects of G-Rg3 pre-treatment on oxidative stress and inflammatory cytokines by HAPE

**FIGURE 3:**
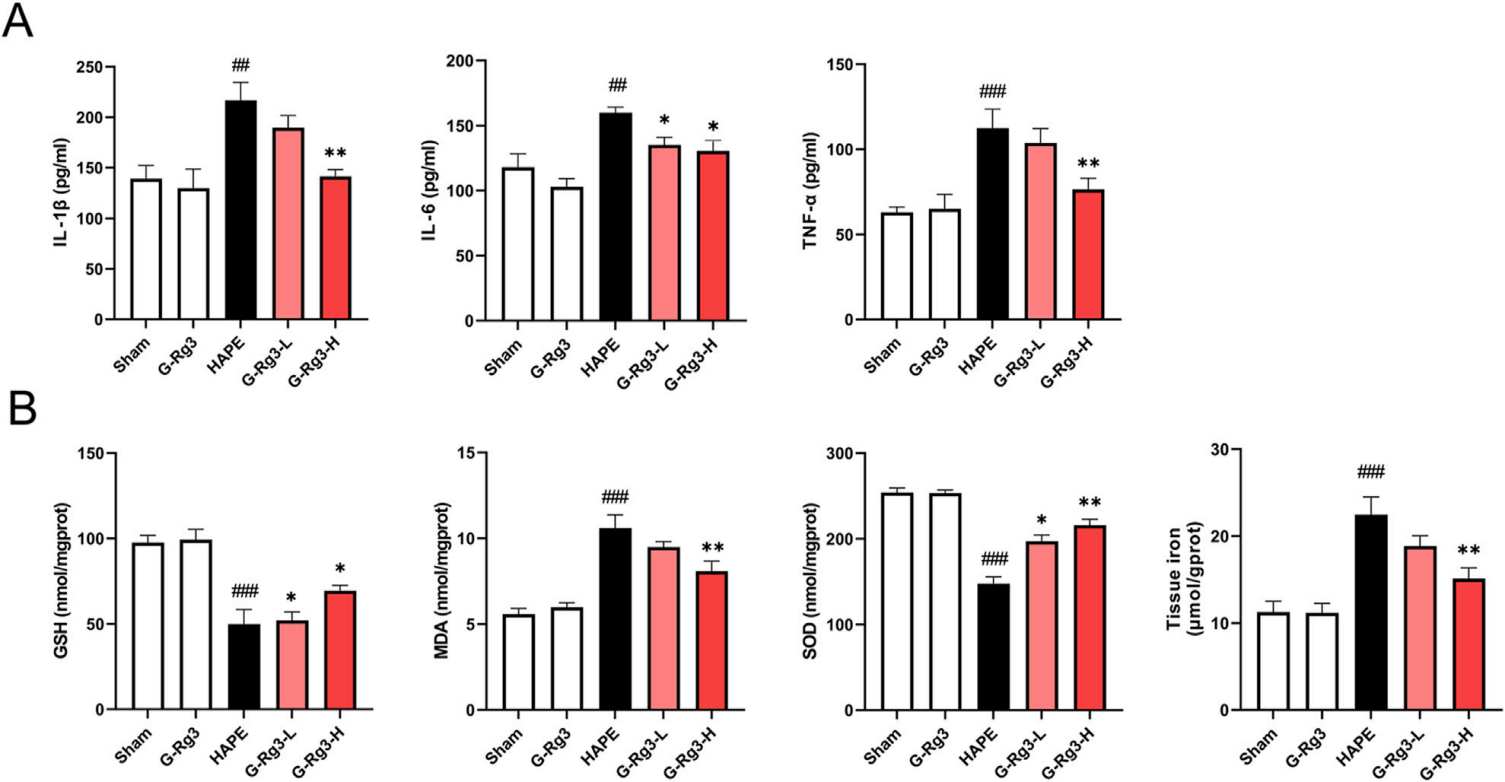
Effects of G-Rg3 pre-treatment on oxidative stress and inflammatory cytokines by HAPE. **(A)** G-Rg3-mediated suppression of proinflammatory cytokines (IL-1β/IL-6/TNF-α). **(B)** Antioxidant regulation through SOD/GSH/MDA biomarkers. Data reflect mean ± SEM (n = 6 biological replicates). One-way ANOVA with Tukey’s *post hoc* analysis: ^##/###^ *P* < 0.01/0.001 vs. Sham controls; ^*/**^ *P* < 0.05/0.01 vs. HAPE group.

HAPE progression is mechanistically linked to acute immune-inflammatory cascade activation, characterized by neutrophilic infiltration, macrophage aggregation, and mediator-induced alveolar-capillary hyperpermeability (Lee et al., 2018; Wang et al., 2022b). Our experimental analyses revealed significant upregulation of pro-inflammatory IL-1β, IL-6, and TNF-α in BALF during hypobaric hypoxia exposure, with prophylactic G-Rg3 administration demonstrating potent cytokine suppression efficacy (Figure 3A). Concomitant evaluation of pulmonary redox homeostasis identified characteristic oxidative imbalance patterns: HAPE-induced samples exhibited heightened lipid peroxidation (elevated MDA), compromised antioxidant defenses (reduced SOD and GSH), and tissue iron overload - all metabolic disturbances effectively normalized through G-Rg3 pre-treatment, as evidenced by complete restoration of baseline oxidative stress markers and iron homeostasis parameters (Figure 3B).

### 3.3 Effects of G-Rg3 pre-treatment on ferroptosis in HAPE

**FIGURE 4:**
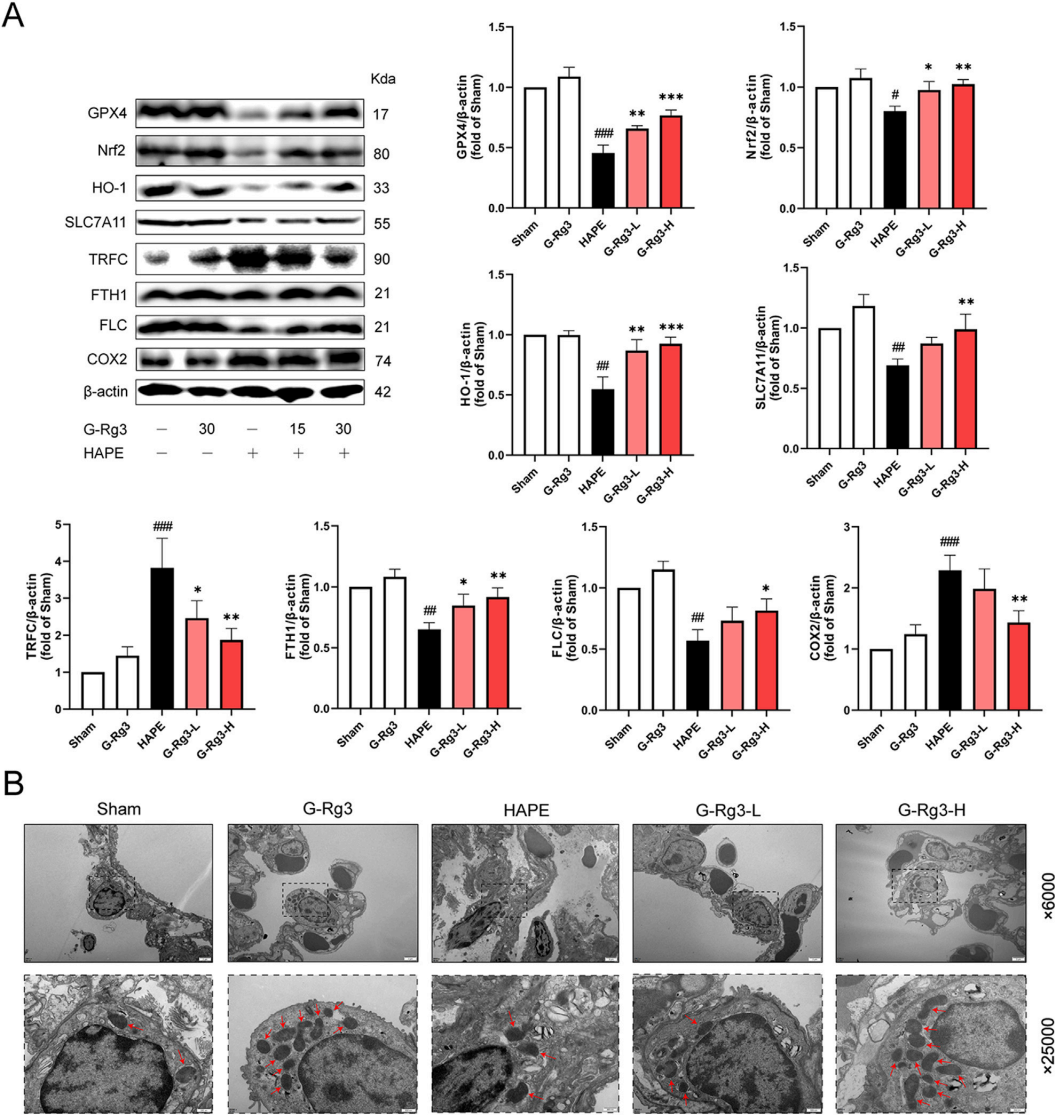
Effects of G-Rg3 pre-treatment on ferroptosis in HAPE. **(A)** Immunoblot quantification of key ferroptotic regulators (GPX4/SLC7A11/FTH1). **(B)** Ultrastructural evidence of mitochondrial pathology in AT2 cells (Red arrows: cristae disruption; scale bars: 2 μm [×6,000], 500 nm [×25,000]). Data represent mean ± SEM (n = 6 biological replicates). One-way ANOVA with Tukey’s *post hoc* analysis: ^#/##/###^ *P* < 0.05/0.01/0.001 vs. Sham; ^*/**/***^ *P* < 0.05/0.01/0.001 vs. HAPE group.

Given that excessive oxidative stress responses can trigger ferroptosis (Djulbegovic and Uversky, 2019), we next investigated the involvement of ferroptosis in HAPE pathogenesis. Western blot analysis indicated that the expression of anti-ferroptosis proteins, including GPX4, Nrf2, HO-1, SLC7A11, FTH1, and FLC, was markedly reduced in model tissues, whereas pro-ferroptosis proteins COX2 and TFRC exhibited elevated expression (Figure 4A). Notably, pre-treatment with G-Rg3 reversed these trends in a dose-dependent manner. To further elucidate the cellular phenotypes linked to ferroptosis, we conducted TEM analysis on lung tissue samples (Figure 4B). Electron microscopic observations revealed characteristic ultrastructural alterations indicative of ferroptosis in type II alveolar epithelial cells from the HAPE group, such as mitochondrial shrinkage, reduced cristae density, and outer mitochondrial membrane damage. These changes were mitigated following G-Rg3 pre-treatment. Based on these findings, we conclude that G-Rg3 pre-administration alleviates HAPE by suppressing ferroptosis.

### 3.4 Network pharmacology prediction and molecular docking analysis of G-Rg3 pre-treatment in HAPE

**FIGURE 5:**
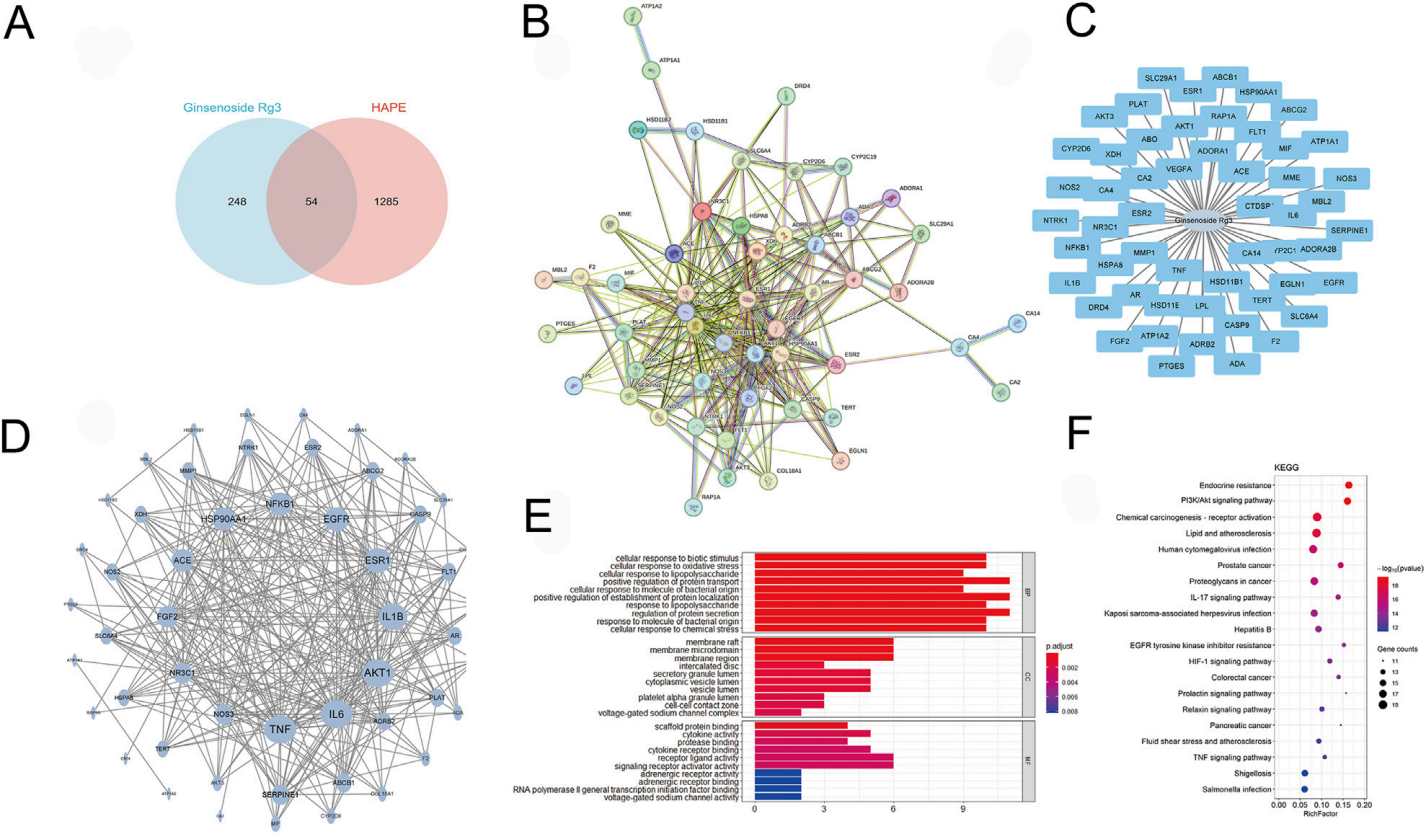
Network pharmacology prediction of G-Rg3 pre-treatment in HAPE. **(A)** Pharmacological target convergence between G-Rg3 and HAPE-associated genes. **(B)** Protein-protein interaction (PPI) network of hub targets, with node size/color intensity reflecting interaction centrality. **(C)** Tripartite network mapping G-Rg3-target-disease-pathway interrelationships. **(D)** High-fidelity subnetwork of critical protein interactions, highlighting topological significance through node degree gradation. **(E)** GO functional annotation categorizing targets into biological processes, molecular functions, and cellular components. **(F)** KEGG pathway enrichment analysis of core therapeutic targets.

Our multi-modal investigation combining network pharmacology and computational modeling revealed G-Rg3’s mechanistic actions against HAPE. Bioinformatic interrogation identified 1,285 HAPE-associated targets and 248 G-Rg3-related genes, with 52 shared candidates forming the core interaction network (Figures 5A–C). Protein interactome mapping prioritized five hub genes (TNF, IL6, AKT1, IL1B, ESR1) through topological centrality analysis (Figure 5D). Functional enrichment demonstrated these mediators coordinate apoptotic regulation (biological process), granular secretory mechanisms (cellular component), and nuclear receptor activation (molecular function), with pathway analysis implicating PI3K/AKT signaling and endocrine resistance as primary therapeutic targets **(** Figures 5E,F).

**FIGURE 6:**
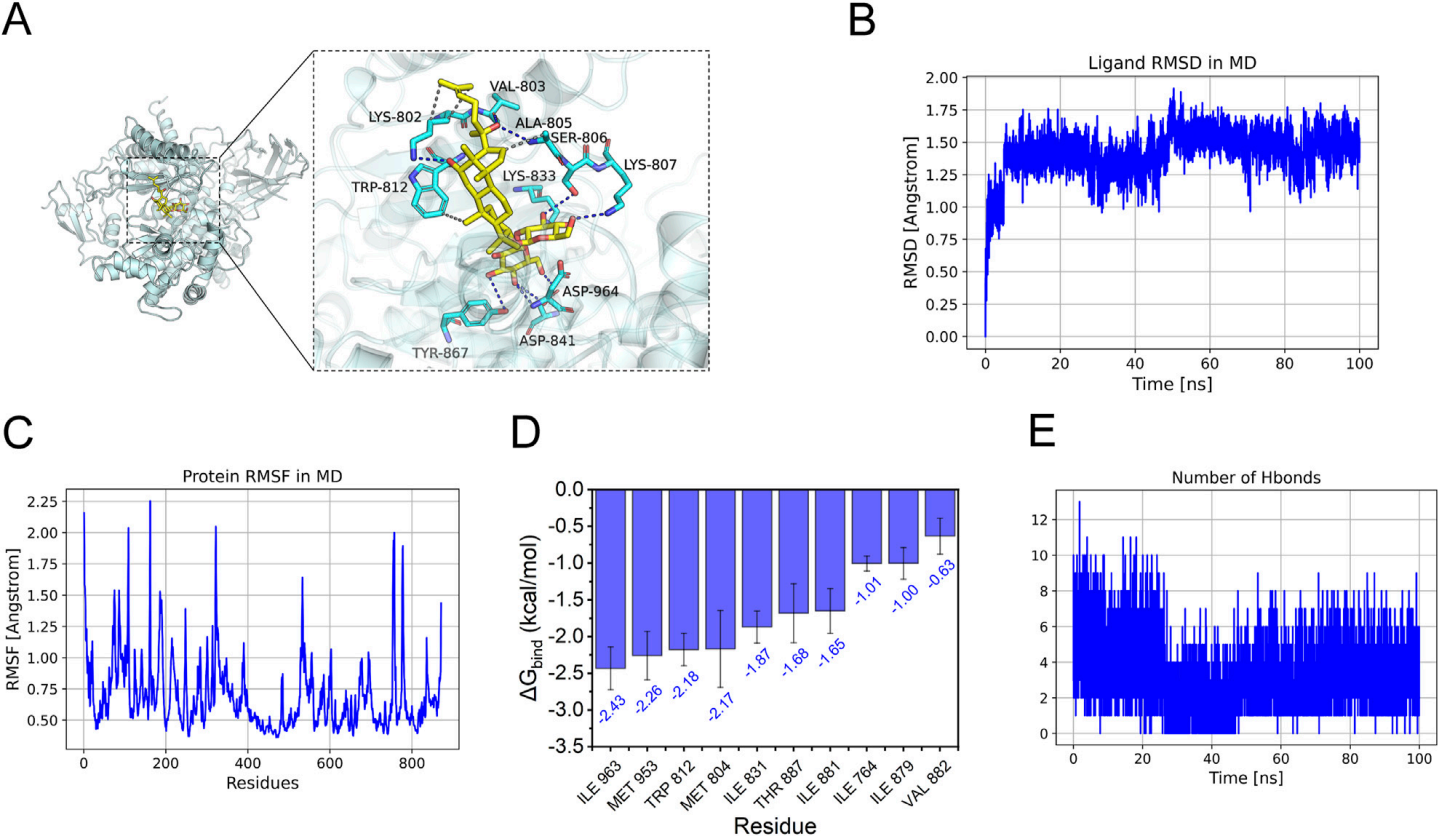
Molecular docking analysis of G-Rg3 pre-treatment in HAPE. **(A)** Molecular docking pose visualization: Gold stick = G-Rg3; Cyan cartoon = PI3K backbone; Blue dashes = hydrogen bonds; Yellow surfaces = hydrophobic interfaces. **(B)** Conformational stability assessment via RMSD. **(C)** Per-residue flexibility mapping (RMSF) with mobile loop regions. **(D)** Binding energy hotspot identification through MM-PBSA decomposition (Top 10). **(E)** Time-dependent hydrogen bond population analysis.

Further molecular docking and dynamics simulations revealed the interaction between Ginsenoside_Rg3 and PI3K. Figure 6A illustrates the binding mode of the PI3K_Ginsenoside_Rg3 complex, demonstrating eight hydrogen bonds (including VAL-803 and LYS-807) and hydrophobic interactions within the active pocket, with a docking energy of −8.48 kcal/mol (Table 1). The 100 ns molecular dynamics simulations confirmed binding stability, showing low RMSD (<2 Å) and RMSF (<2 Å) values (Figures 6B,C) (Chen et al., 2024). MM-GBSA calculations yielded a binding energy of −34.10 ± 3.10 kcal/mol (Table 2), primarily driven by van der Waals and electrostatic interactions. Ten key residues (including TRP-812 and ILE-963) contributed significantly to binding (Figure 6D). Sustained hydrogen bonding (4 bonds/frame on average, Figure 6E) validated the high-affinity binding potential of this interaction.

**TABLE 1:** Scores for the binding affinity of the complexes.

Target_name	Ligand_name	Docking_score (kcal/mol)
3ml9-PI3K	Ginsenoside_Rg3	−8.484

**TABLE 2:** MM/GBSA predictions for binding free energies and energy components (kcal/mol).

System name	PI3K/Ginsenoside_Rg3
Δ*E*_vdw_	−58.96 ± 2.20
Δ*E*_elec_	−38.11 ± 2.68
ΔG_GB_	71.67 ± 5.68
ΔG_SA_	−8.70 ± 0.68
ΔG_bind_	−34.10 ± 3.10

ΔE_vdW_: van der Waals energy.

ΔE_elec_: electrostatic energy.

ΔG_GB_: electrostatic contribution to solvation.

ΔG_SA_: non-polar contribution to solvation.

ΔG_bind_: binding free energy.

According to the findings from network pharmacology and docking analysis, G-Rg3 might mitigate HAPE and ferroptosis by stimulating the PI3K/AKT signaling pathway via strong affinity binding with PI3K, which was needed additional experimental validation.

### 3.5 G-Rg3 ameliorates HAPE by inhibiting ferroptosis through activation of the PI3K/AKT pathway

**FIGURE 7:**
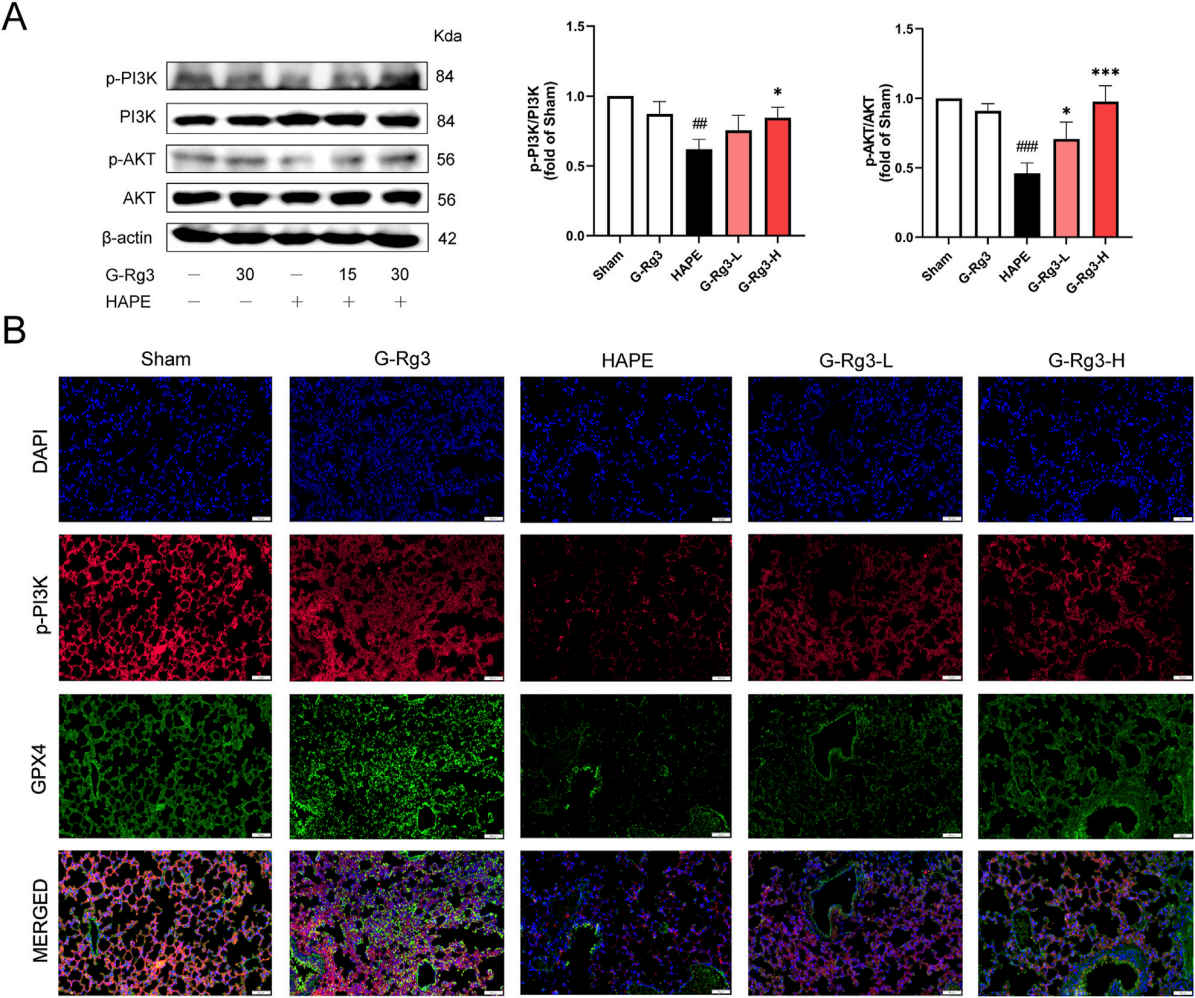
G-Rg3 ameliorates HAPE by inhibiting ferroptosis through activation of the PI3K/AKT pathway. **(A)** Immunoblot quantification of PI3K, p-PI3K, AKT, and p-AKT. **(B)** Confocal microscopy of merged p-PI3K (green)/GPX4 (red) fluorescence signals (scale bar: 20 μm, ×400). Data represent mean ± SEM (n = 6 biological replicates). One-way ANOVA with Tukey’s *post hoc* analysis: ^##/###^ *P* < 0.01/0.001 vs. Sham; ^*/***^ *P* < 0.05/0.001 vs. HAPE.

To confirm the predictions of network pharmacology through biochemical approaches, we conducted Western blot and immunofluorescence analyses. The Western blot results showed that in mice exposed to high-altitude hypobaric hypoxia-induced HAPE, the phosphorylation levels of PI3K and AKT were substantially decreased. Nevertheless, G-Rg3 pre-treatment markedly inhibited the reduction in phosphorylation levels of PI3K and AKT (Figure 7A). Immunofluorescence analysis further indicated that in the HAPE group, the co-localization between PI3K activation and GPX4 antioxidant enzyme expression was inhibited. In contrast, prophylactic treatment with G-Rg3 successfully restored the expression of these two biomarkers (Figure 7B). Taken together, these findings suggest that G-Rg3 can effectively activate the PI3K/AKT signaling pathway.

### 3.6 G-Rg3 pre-treatment inhibition of ferroptosis was dependent on the PI3K/AKT pathway

**FIGURE 8:**
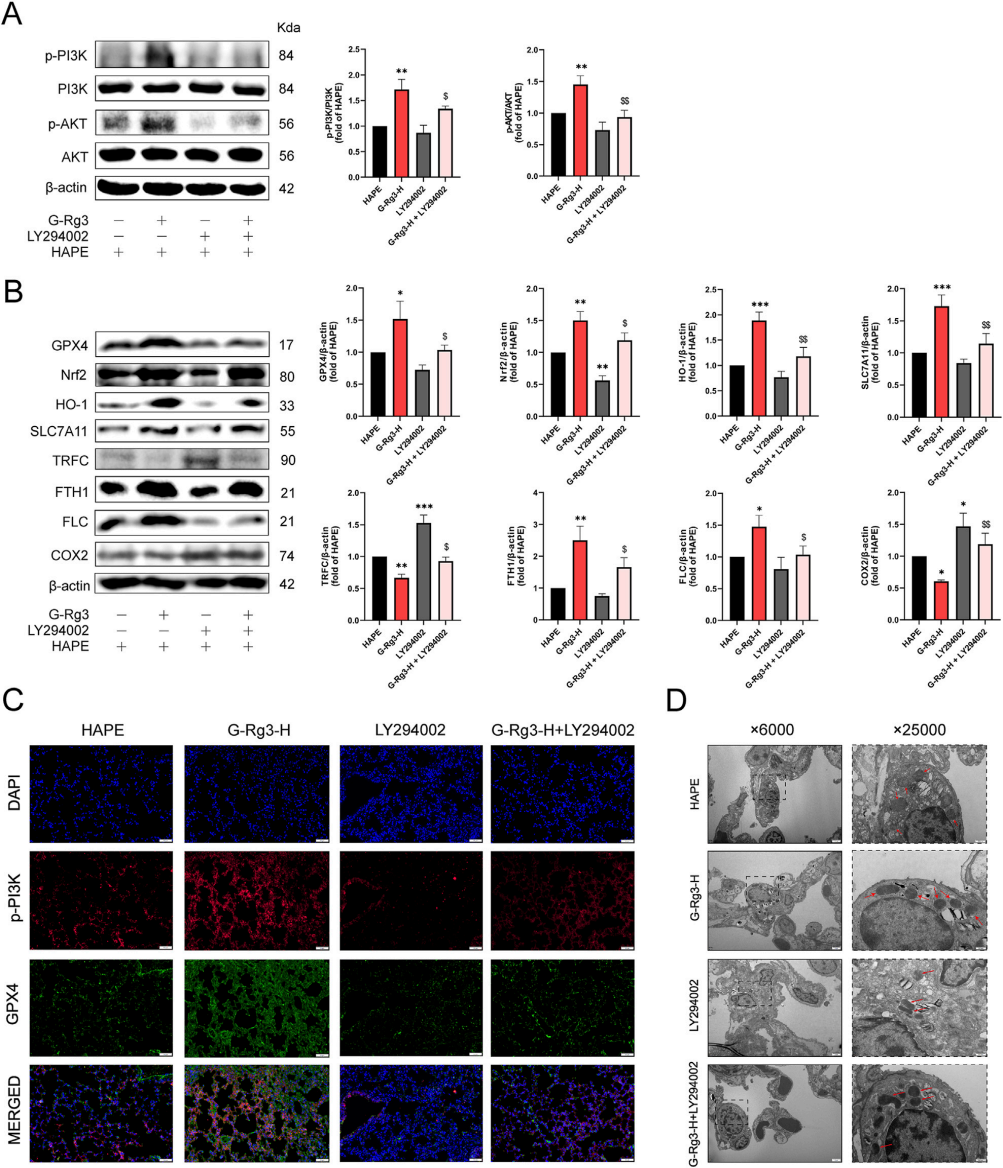
G-Rg3 pre-treatment inhibition of ferroptosis was dependent on the PI3K/AKT pathway. **(A)** Immunoblot quantification of PI3K, p-PI3K, AKT, and p-AKT. **(B)** Ferroptosis biomarker analysis. **(C)** Confocal microscopy of p-PI3K (green, Alexa Fluor 488) and GPX4 (red, Cy3) co-localization in alveolar epithelia (scale bar: 20 μm; ×400). **(D)** Ultrastructural ferroptosis markers: mitochondrial cristae dissolution (red arrows) in type II alveolar epithelial cells (scale bars: 2 μm [×6,000]; 500 nm [×25,000]). Data represent mean ± SEM (n = 6 biological replicates). One-way ANOVA with Tukey’s *post hoc* analysis: ^*/**/***^ *P* < 0.05/0.001 vs. HAPE, ^$/$$^ *P* < 0.05/0.01 vs. G-Rg3-H group.

To determine whether the inhibitory action of G-Rg3 on ferroptosis in a HAPE mouse model is dependent on the activation of the PI3K/AKT signaling pathway, this study utilized the specific PI3K inhibitor LY294002. Western blotting analysis demonstrated G-Rg3’s capacity to enhance PI3K/AKT phosphorylation, an effect abrogated by concurrent LY294002 administration (Figure 8A). Subsequent ferroptotic biomarker profiling revealed G-Rg3’s dual regulatory function: coordinated upregulation of GPX4, Nrf2, HO-1, SLC7A11, and ferritin complexes (FTH1/FLC) concurrent with suppression of TFRC/COX2 expression (Figure 8B). Crucially, the inhibitor LY294002 negated these modulatory effects. Complementary validation through immunofluorescence and transmission electron microscopy corroborated the molecular analyses, demonstrating restored subcellular architecture and antioxidant enzyme localization (Figures 8C,D). These findings mechanistically establish PI3K/AKT signaling as the principal conduit mediating G-Rg3’s anti-ferroptotic efficacy in HAPE pathophysiology.

### 3.7 The protective effect of G-Rg3 pre-treatment on HAPE was mediated through the PI3K/AKT signaling pathway

**FIGURE 9:**
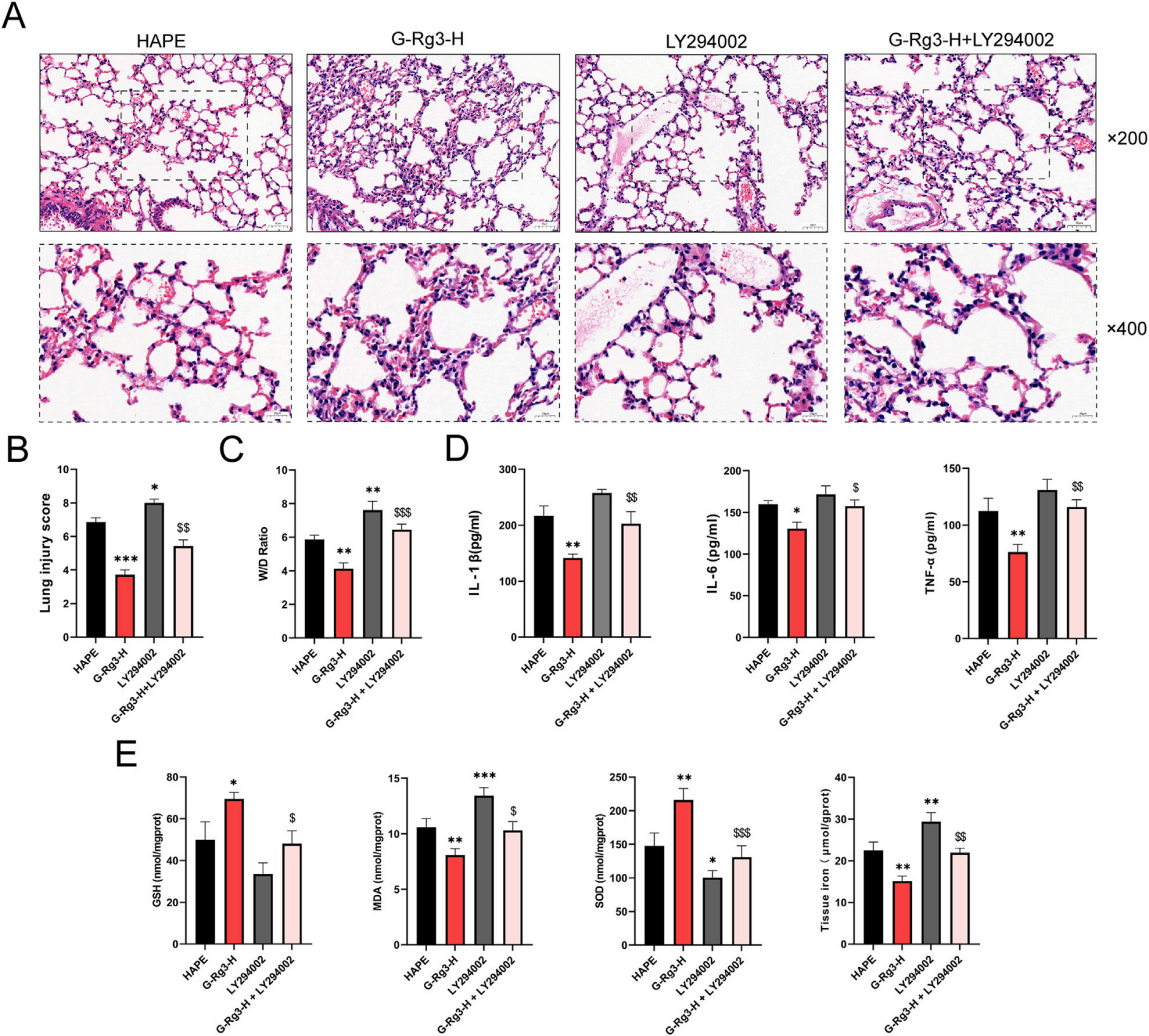
The protective effect of G-Rg3 pre-treatment on HAPE was mediated through the PI3K/AKT signaling pathway. **(A)** H&E-stained lung sections (upper: ×200, 50 μm scale; lower: ×400, 20 μm scale). **(B)** Semi-quantitative histopathological assessment of HAPE. **(C)** Pulmonary edema quantification (W/D ratio). **(D)** Analysis of the effect of LY294002 on inflammatory markers (IL-1β, IL-6, and TNF-α) in BALF. **(E)** Evaluation of LY294002’s influence on antioxidant enzymes (GSH, SOD) and oxidative stress markers (MDA) as well as tissue iron levels in lung tissues. Data represent mean ± SEM (n = 6 biological replicates). One-way ANOVA with Tukey’s *post hoc* analysis: ^*/**/***^ *P* < 0.05/0.001 vs. HAPE, ^$/$$/$$$^ *P* < 0.05/0.01 < 0.001 vs. G-Rg3-H group.

Histopathological evaluation of LY294002-treated lung tissues revealed pronounced structural damage characterized by inflammatory infiltration, alveolar wall thickening, and intra-alveolar congestion (Figure 9A). These pathological changes were corroborated through semi-quantitative scoring and edema quantification (Figures 9B,C). G-Rg3 pre-treatment notably mitigated these alterations, restoring near-normal pulmonary architecture. Concurrent analysis demonstrated LY294002-induced upregulation of inflammatory mediators and oxidative stress markers, effects substantially attenuated by G-Rg3 administration (Figures 9D,E). This integrated analysis confirms PI3K/AKT signaling modulation as the central mechanism underlying G-Rg3’s therapeutic efficacy against HAPE-associated pulmonary injury.

## 4 Discussion

Recent years have witnessed increased human activity in high-altitude regions, amplifying clinical demands for managing hypoxia-related cardiopulmonary disorders. Particularly concerning is the prevalence of HAPE. Our study employed an HAPE mouse model to systematically evaluate the therapeutic potential of G-Rg3. Pretreated mice exhibited markedly attenuated HAPE-associated pathological features, concurrent with normalized HIF-1α overexpression and robust anti-inflammatory/antioxidant responses. Further mechanistic investigations identified ferroptosis suppression as a critical component of G-Rg3’s action. Central to this protective effect is the metabolite’s activation of PI3K/AKT signaling, as evidenced by integrated network pharmacology analyses, computational modeling, and targeted pathway inhibition experiments. Investigations reveal that the traditional botanical metabolite G-Rg3 holds promise for high-altitude HAPE treatment through its multi-action regulatory effects. By targeting acute pathological changes and alleviating hypoxia-induced secondary injuries, it offers significant pharmacological insights supporting the clinical translation of plant-based therapies.

The complex physiological demands imposed by high-altitude hypoxia present significant challenges. A key mechanism driving the development of HAPE involves the deregulation of HIF-1α and VEGF (Wang et al., 2022b). Under conditions of reduced pressure and oxygen availability, cells initiate an adaptive response by stabilizing HIF-1α, which subsequently triggers elevated VEGF expression. Both factors are crucial in the progression of HAPE. Studies have shown that hypoxic conditions amplify the activity of HIF-1α, positioning it as the principal regulator of genes responsive to hypoxia—genes that are vital for cellular survival. In pathological scenarios, the heightened induction of VEGF, mediated by HIF-1α, fosters irregular angiogenesis and accelerates disease progression. Our experimental data corroborate these findings, indicating that increased levels of HIF-1α and VEGF intensify the pathological processes associated with pulmonary edema. Importantly, this investigation expands on earlier studies (Ahmmed et al., 2019; Lv et al., 2023) by illustrating the suppressive impact of G-Rg3 on the expression levels of HIF-1α and VEGF in HAPE models.

Simultaneously, pro-inflammatory cytokines such as TNF-α, IL-1β, and IL-6 have been identified as pivotal factors influencing the dynamic changes in white blood cells during HAPE, which play critical roles in regulating the proliferation, migration, and differentiation of immune cells (Tanaka et al., 2014) In mouse models, the systemic levels of these cytokines were significantly elevated following HAPE induction. These observations align with previous reports associating cytokine storms with inflammatory lung injury (Arya et al., 2013). The ability of G-Rg3 to modulate these pathways underscored its dual therapeutic potential, illustrating its influence on the inflammatory cascade within the pathophysiology of HAPE.

Hypobaric hypoxia in HAPE induces mitochondrial dysfunction and excessive ROS generation (superoxide, hydrogen peroxide), driving oxidative damage and depleting antioxidant reserves (Irarrázaval et al., 2017). his oxidative cascade triggers membrane lipid peroxidation, quantified by elevated MDA levels (Weismann et al., 2011), while concurrently suppressing SOD activity which is a critical antioxidant enzyme (Wang et al., 2018)). The hypoxia-induced inhibition of system Xc^−^ (SLC7A11/SLC3A2) further diminishes GSH synthesis through cysteine deprivation, impairing both ROS neutralization and GPX4-mediated ferroptosis prevention (Endale et al., 2023; Mailloux et al., 2013). Our findings reveal a pathological feedback loop in HAPE: diminished SOD activity facilitates superoxide accumulation, heightened MDA concentrations signify accelerated lipid peroxidation, and GSH depletion undermines antioxidant mechanisms, all of which contribute to pulmonary edema. Importantly, pre-treatment with G-Rg3 can counteract these disruptions, restore SOD function and GSH levels in lung tissue, and lower MDA, effectively interrupting this disease progression pathway.

Emerging evidence identifies oxidative stress-mediated iron metabolism dysregulation and ferroptosis as pivotal mechanisms in HAPE. The pathogenic triad of ROS accumulation, lipid peroxidation, and iron overload which established contributors to pulmonary disorders, is exacerbated under hypoxic conditions (Wang et al., 2024). Mechanistically, hypoxia inhibits Nrf2, suppressing key iron regulators (SLC7A11, HO-1, FTH1) and weakening antioxidant defenses (Loboda et al., 2016; Su et al., 2025; Zhang S. et al., 2025). This suppression triggers two detrimental pathways: 1) impaired cystine transport through System Xc^−^ disrupts glutathione synthesis and GPX4 antioxidant activity; 2) reduced HO-1/FTH1 expression enhances free iron toxicity via Fenton reactions (Basiouny et al., 2025; Fu et al., 2022; Qian et al., 2024). Concurrent elevation of ferroptosis markers TFRC and COX2 further drives pathological iron uptake and inflammatory lipid damage (Liu M. J. et al., 2025; Zhuang et al., 2024). Our experimental data validate this mechanistic model in HAPE progression. Diseased mice exhibited characteristic ferroptosis signatures: depressed anti-ferroptosis proteins (GPX4, Nrf2, HO-1, SLC7A11, FTH1/FLC) alongside elevated TFRC, COX2, and tissue iron. Crucially, G-Rg3 treatment ameliorated these imbalances, reactivating ferroptosis-suppressing pathways and restoring iron homeostasis. This dual antioxidant-iron regulatory capability positions G-Rg3 as a promising therapeutic agent targeting the oxidative-ferroptosis axis in HAPE.

The PI3K/AKT signaling axis (Liu et al., 2025c) functions as a master regulator of cellular homeostasis, coordinating processes from metabolic regulation to survival pathways (Huang et al., 2022). Mechanistically, PI3K initiates signaling by phosphorylating membrane phosphatidylinositol to generate PIP3, which facilitates AKT membrane recruitment and subsequent activation through phosphorylation events. Activated AKT then orchestrates downstream biological responses through its effector network (Vanhaesebroeck et al., 2010). This pathway exhibits dual ferroptosis-inhibitory capacities: (1) SREBP1-mediated lipogenesis enhancement reduces membrane phospholipid peroxidation susceptibility (Zheng et al., 2024); (2) AKT-driven Nrf2 phosphorylation (Ser40) promotes KEAP1 dissociation, enabling nuclear translocation to boost antioxidant defenses (glutathione synthesis) and iron regulatory capacity (Cheng et al., 2023). Our integrated pharmacological investigation reveals G-Rg3’s therapeutic mechanism through PI3K/AKT modulation. Network pharmacology prioritized this pathway as the prime HAPE intervention target, corroborated by computational modeling showing stable G-Rg3-PI3K binding through hydrogen bonds and hydrophobic interactions. Immunoblot analyses demonstrated G-Rg3’s capacity to restore depressed PI3K/AKT phosphorylation in HAPE lungs. Crucially, co-treatment with LY294002 (PI3K inhibitor) abolished G-Rg3’s protective effects against both inflammatory cytokines and ferroptosis markers (GPX4/HO-1 downregulation), conclusively validating pathway centrality.

This study identified G-Rg3 as a selective PI3K/AKT activator that can alleviate ferroptosis in high-altitude pulmonary edema (HAPE) by coordinating the regulation of lipid metabolism, iron homeostasis, and oxidative defense. Four limitations need to be addressed when establishing the therapeutic framework of G-Rg3: (1) The limitations of the acute hypobaric model require gradient altitude studies that simulate the progression of human HAPE; (2) Further *in vitro* studies are needed to clarify the mechanism by which G-Rg3 prevents HAPE; (3) Orthogonal validation through gene editing, co-immunoprecipitation target identification, and surface plasmon resonance is necessary for the confirmation of the PI3K-based pathway; (4) GLP-compliant toxicological analyses (chronic toxicity, tissue-specific pharmacokinetics) are crucial for clinical translation. These further studies will provide a more solid theoretical basis for G-Rg3-based intervention strategies for HAPE.

## 5 Conclusion

**FIGURE 10:**
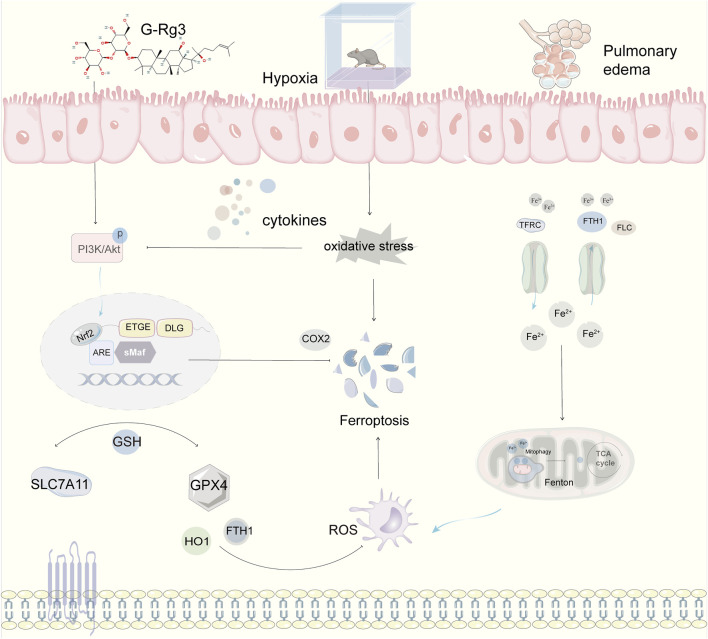
Mechanistic Framework of G-Rg3-Mediated Pulmonary Protection in Murine HAPE Models. Pharmacological preconditioning with G-Rg3 (30 mg/kg, i. p.) ameliorates hypoxia-induced pulmonary edema through coordinated ferroptosis-PI3K/AKT axis modulation.

This research highlights that the active constituent of ginseng, G-Rg3, can successfully mitigate acute high-altitude pulmonary edema triggered by hypobaric hypoxia. This effect is achieved through the activation of the PI3K/AKT signaling pathway and the cooperative modulation of inflammatory responses, oxidative stress, and ferroptosis (Figure 10). Importantly, the therapeutic timing for G-Rg3 includes preventive administration up to 72 h prior to ascent, with optimal efficacy-to-dose performance observed at a dosage of 30 mg/kg. Beyond counteracting the HIF-driven inflammatory-oxidative cascade, focusing on the PI3K/AKT regulatory axis offers a molecular foundation for designing combination therapies. These underlying mechanisms indicate that G-Rg3 could serve both as a standalone treatment in high-altitude emergency care and leverage its distinctive iron homeostasis regulation capabilities for managing chronic high-altitude illnesses or systemic inflammation-related hypoxic conditions. This provides a comprehensive approach for advancing traditional medicinal plants into modern applications.

## Data Availability

The original contributions presented in the study are included in the article/supplementary material, further inquiries can be directed to the corresponding authors.
